# Does Pet Arrival Trigger Prosocial Behaviors in Individuals with Autism?

**DOI:** 10.1371/journal.pone.0041739

**Published:** 2012-08-01

**Authors:** Marine Grandgeorge, Sylvie Tordjman, Alain Lazartigues, Eric Lemonnier, Michel Deleau, Martine Hausberger

**Affiliations:** 1 CHRU de Brest, Hôpital de Bohars, Centre de Ressources Autisme, Bohars, France; 2 UMR-CNRS 6552, Laboratoire Ethologie Animale et Humaine, Rennes, France; 3 CHRU Guillaume Régnier, Rennes, France; 4 Centre de recherches en psychologie, cognition et communication, Rennes, France; Boston College, United States of America

## Abstract

Alteration of social interactions especially prosocial behaviors – an important aspect of development – is one of the characteristics of autistic disorders. Numerous strategies or therapies are used to improve communication skills or at least to reduce social impairments. Animal-assisted therapies are used widely but their relevant benefits have never been scientifically evaluated. In the present study, we evaluated the association between the presence or the arrival of pets in families with an individual with autism and the changes in his or her prosocial behaviors. Of 260 individuals with autism - on the basis of presence or absence of pets - two groups of 12 individuals and two groups of 8 individuals were assigned to: study 1 (pet arrival after age of 5 *versus* no pet) and study 2 (pet *versus* no pet), respectively. Evaluation of social impairment was assessed at two time periods using the 36-items ADI-R algorithm and a parental questionnaire about their child-pet relationships. The results showed that 2 of the 36 items changed positively between the age of 4 to 5 (t_0_) and time of assessment (t_1_) in the pet arrival group (study 1): “offering to share” and “offering comfort”. Interestingly, these two items reflect prosocial behaviors. There seemed to be no significant changes in any item for the three other groups. The interactions between individuals with autism and their pets were more – qualitatively and quantitatively - reported in the situation of pet arrival than pet presence since birth. These findings open further lines of research on the impact of pet’s presence or arrival in families with an individual with autism. Given the potential ability of individuals with autism to develop prosocial behaviors, related studies are needed to better understand the mechanisms involved in the development of such child-pet relationship.

## Introduction

Impairments of social development associated with communication deficits, restricted interests and repetitive behaviors constitute the triad of autistic disorders [1], [2]. Individuals with autism have difficulty interacting with others as well as using and interpreting nonverbal communication. Social impairments have been regarded as primary deficits by several authors [3], [4] since they are among the first symptoms of autistic disorders (e.g. difficulty in participating in imitative or pretend play [5], [6]). Individuals with autism appear to have problems recognizing, understanding and expressing both feelings and intentions, which may be due to a lack of “theory of mind” [7]. These individuals fail to infer mental states and display impairment of abilities to understand and manage emotions (i.e. understand the other's feelings and display appropriate behavior or response [4], [8]).

Many strategies, supports or therapies have been aimed at improving the everyday lives and social interactions of individuals with autism [9], [10] For example, peer-mediated interventions have proved to be useful through increasing the communicative interactions and stimulating the development of joint attention [11]. Complementary and alternative interventions are also proposed: relaxation, music or activities with animals [12]. Indeed, since early findings by Levinson’s reporting that a dog could help in therapy [13], animal assisted therapies (AAT) have been used largely. Sessions with dogs, horses or dolphins are proposed, and considered overall as beneficial to improve prosocial behaviors [14]–[17]. However, to date, there is no scientific evaluation of their relevant benefit [18], [19]. Moreover, the context in which AAT occur must be accounted for. The impact of having a pet in a therapeutic or home setting seems to be different when encountering humans [20].

More broadly, beneficial effects of having a pet at home have been reported for improvement of health or well-being of elderly, isolated women, adults and children [21]–[26]. It is considered as a source of non judgmental and positive affection [27], [28]. Several studies suggest that children learn prosocial behaviors through their interactions with pets [29]–[31]. These prosocial behaviors constitute an important aspect of a child’s development. They are triggered by pet’s presence under certain circumstances (e.g. if a strong bond is formed, if the pet lives at home or if the human partner is younger than 6 years old [32]–[34]). Thus, bonding with a pet may help with developing some prosocial behaviors. This hypothesis seems to be consistent with the results of other studies about the reciprocal behavior that leads an animal to exceptional learning (e.g. Alex the parrot [35], Hoover the seal [36], Kanzi the chimpanzee [37]).

In the present study, we hypothesized that a pet at home might help individuals with autism to develop some prosocial behaviors. For this, we compared three situations: never owned a pet, owned a pet since birth (i.e. pet has been part of the individual’s environment) or owned a pet after the age of 5. The age of 4 to 5 is considered as a “key age” in autistic disorders [38] because it seems to be representative of the period when the severity of autism is the most important. Indeed, older subjects might outgrow some of the major impairments. Accordingly, there is a need to avoid focusing on the basis of behavior in childhood. Consequently, the Autism Diagnostic Interview-Revised (ADI-R) explains that the most satisfactory compromise is to consider the age of 4 to 5 as the key age to evaluate the individual’s behavior.

The arrival of a pet in a family has been shown to increase the level of interactions between family members: they spend more time together and share joint attention on the new family member [39]. The new arrival of a pet potentially elicits more attention in individuals with autism thus leading to a greater chance of bonding with the pet. We further hypothesized that the arrival of a pet when the human partner was old enough to “realize this change” would increase the chances of improving the human’s prosocial behaviors. For this, we evaluated the individual’s impairments using the ADI-R [38], to compare two time periods (i.e. t_0_ at the age of 4 to 5 and t_1_ at the time of assessment), and a parental questionnaire about the child-pet relationship. Since direct questioning of individuals with autism can be complicated, we only used parental reports in this study. According to the literature, individuals with autism display delays and deficits in the acquisition of language (e.g. complete absence of functional communication, impairments in conversation) [40], [41]. Parents are a reliable source of information in regard to the evaluation of their child’s developmental problems [42], [43]. For example, in a previous study, Siegel et al. [44] found that parental reports about typical daily behaviors of their children with autism confirmed observations made during diagnostic play sessions by trained professionals. In addition, parental reports concerning both their pets and their child's behaviors are more reliable than children's interviews [45].

## Methods

### Participants

All the individuals with autism (n = 260; 59♀/191♂; mean age, 15±7.5 years old, range from 6 to 34 years old) in this study, came from the “*Centre de Ressources sur l’Autisme de Bretagne*” (Bohars, France) or the child day-care facilities controlled by the Bicêtre and Reims University Hospitals (France). The cognitive and behavioral assessments were approved by the ethics committee of Bicêtre hospital (the committee was not specific to this study). It is worth mentioning that the present research was non-invasive and did not involve pharmacological interventions. Hence, in accordance to the ethics committee, parents (or guardians) gave a simple verbal consent. All individuals met DSM-IV criteria for autistic disorders [1]. As part of a routine follow-up of individuals with autism, the same psychiatrists did the diagnosis and the ADI-R [38] assessment to confirm the diagnosis.

### Cognitive and Behavioral Assessments

The cognitive functioning of individuals with autism from child day-care facilities of the University Hospitals of Bicêtre and Reims (n = 70) was assessed by two psychologists using the age-appropriate Weschler intelligence scale and the Kaufman K-ABC [46]. All assessed individuals with autism were cognitively impaired (mean full scale IQ ± S.D: 42.1±3.4, with a range of 40–58; mean verbal IQ ± S.D: 45.2±2.3, with a range of 45–57; mean performance IQ ± S.D: 45.2±4.4, with a range of 45–80).

ADI-R was used to assess the behavior of 260 participants with autism [38]. ADI-R, an extensive, semi-structured parental interview, was conducted by trained psychiatrists (EL, ST). The structuring lies in the details of the predetermined codings for each behavioral item. The interview schedule specifies a variety of screening questions, the purpose of which is to guide the interviewer on the content of the response (yes or no responses from the informant, i.e. parents or guardians, were inadequate). Behavioral descriptions are coded. The codings have been devised with the aim of differentiating developmental delay from deviance. Thus, for each section of the interview, there is an initial compulsory probe printing. The interviewer should then continue to ask further questions until he/she is able to make the coding for each item, for example, using different supplementary probes proposed in the ADI-R. The ADI-R scale assessed the three major domains of autistic impairments: (1) reciprocal social interactions, (2) verbal and non-verbal communication and (3) stereotyped behavior and restricted interests. The presence of verbal language is defined as daily, functional and comprehensible use of spontaneous phrases of at least three words, including at least sometimes, a verb [38].

The ADI-R algorithm is validated to assess the behavior and is based on the 4-to-5-year-old period of life. To reveal possible variations, we compared the ratings at the current period (t_1_) of the subset of ADI-R to those at the age of 4 to 5 (t_0_) [47]. The severity of behavioral impairments was scored using the subset of ADI-R items included in the ADI-R algorithm, following the procedure previously described [48]. We give below the mean score for each main domain: (1) total reciprocal social interaction (15 items), (2) total verbal communication and total non-verbal communication (13 items for non verbal patients, the score was based on 9 items), (3) total stereotypies (8 items). A score for the combined domain (social/communication/stereotypies) was calculated and regarded as a global score of autism severity (Table 1).

**Table 1:** Demographic and behavioral characteristics of study groups (G_0A_ and G_0B_ never owned pet; G_alw_ always owned a pet; G_pet_ didn’t own a pet before the age of 5, but owned at least one at the time of assessment).

	G_OA_ (n = 12)	G_pet_ (n = 12)	G_OB_ (n = 8)	G_alw_ (n = 8)
**Gender** (M/F)	9/3	9/3	4/4	4/4
**Age** (months; mean ± SD; range)	122.8±52.3 (87–180)	137.1±60.6 (80–185)	137.2±42.7 (73–201)	128.6±44.4 (75–200)
**Overall level of language** 1	9/3	9/3	2/6	2/6
**Epilepsy** (yes/no)	8/0	8/0	1/11	0/12
**ADI-R at t_0_** (mean ± SD)				
***Total***	44.5±5.3	44.6±4.5	45.8±1.7	43.1±3.0
*Reciprocal social interactions*	23.2±3.1	22.1±3.6	25.0±1.9	21.9±2.1
*Non verbal Communication*	10.3±2.2	11.0±1.7	10.9±0.6	9.9±1.5
*Verbal Communication* 2	15.9±2.6	16.9±1.0	17.3±0.6	13.5±1.8
*Restricted and repetitive behaviors*	5.4±1.1	5.6±0.9	7.8±1.3	9.9±1.0
**ADI-R at t_1_** (mean ± SD)				
***Total***	38.7±4.5	38.6±5.0	39.8±7.0	38.8±5.9
*Reciprocal social interactions*	22.1±3.6	18.8±3.5	18.6±4.5	19.1±3.6
*Non verbal Communication*	7.1±2.0	10.3±1.9	10.3±2.9	7.5±2.8
*Verbal Communication* 2	11.5±1.9	14.9±1.5	14.5±2.0	10.4±2.2
*Restricted and repetitive behaviors*	5.1±1.2	4.8±0.9	6.6±1.4	9.3±1.5
**Mann Whitney U-test at t_0_**	**U**	**p-value**	**U**	**p-value**
***Total***	152	0.931	81.5	0.170
*Reciprocal social interactions*	157	0.707	81	0.189
*Non verbal Communication*	138.5	0.521	74	0.564
*Verbal Communication* 2	133.5	0.353	72	0.554
*Restricted and repetitive behaviors*	148	0.931	74	0.560

1 Absence/presence of verbal language as defined according to the ADI-R criteria.

2 Scores corresponded to children who had a verbal language according to the ADI-R criteria.

Based on direct clinical observation for each participant by an independent psychiatrist, a diagnosis of autistic disorder was made according to DSM-IV [1] and ICD-10 [49] criteria and was confirmed by the ADI-R ratings. We didn’t perform an Autistic Diagnostic Observation Schedule [50] assessment. It has not been a routine practice in France before 2008 [51].

### Questionnaires on Human-pet Relationships

Parents were interviewed by phone by one of the investigators (MG) not involved in the ADI-R scoring (i.e. was not aware of the data values). They were asked to answer a short standardised questionnaire about the child-pet relationship. No further information was given before the beginning of the questionnaire. Verbal informed consent was given by the parents (or guardians) when the questionnaire on human-pet relationships was filled in. The consent form explained that the questionnaire and ADI-R data will be used together. ADI-R evaluation was performed by the psychiatrist who was not aware of our project. Therefore, neither parents nor evaluators were influenced by the potential expectations of the pet’s impact. The interval between the ADI-R assessment and the questionnaire phase was less than one year. The data from parental questionnaire were collected between winter 2006 and winter 2007.

The questionnaire was about the presence (or absence) of pets in the family at t_0_ (i.e. at the individual’s age of 4 to 5) and at t_1_ (i.e. at the time of ADI-R assessment). If one or more pets were present, parents gave information on the species and the pet ownership duration, as well as their child-pet relationship. The following data were gathered (yes or no answers): tactile interactions, visual interactions, play, care (*e.g.* feeding, walking with the pet, brushing the pet), time spent with and any privileged relationship. The above data helped us to evaluate the individual-pet bond. Moreover, parents specified whether the pet was specially acquired for their child with autism. Pets were dogs, cats and/or little furry animals. Half of the pets were acquired for the individuals with autism.

### Study 1: Arrival of a Pet between the Age of 4 to 5 and the Time of ADI-R Assessment

From the initial pool of 260 participants, we selected two groups. The first group, G_pet,_ did not own a pet before t_0_ but owned at least one afterwards (n = 12; pets were dogs, cats and one hamster). The G_pet_ individuals were matched with control individuals – who never owned a pet (G_0A_, n = 12) - for sex, age, overall level of language (absence/presence of verbal language as defined by ADI-R criteria in the following section) and history of epilepsy (Table 1; all chi-square tests and Mann Whitney U-tests p>0.05). Both the total score and the sub-scores of the ADI-R were not significantly different (all Mann-Whitney U-tests, p>0.05; Table 1). The G_pet_ and G_0A_ mean age was 10.8±2.3 years old at t_1_. On the average, we obtained the G_pet_ parents responses to the questionnaire 79±29 months after the pet's arrival.

### Study 2: Owned a Pet since Birth

We investigated whether the arrival (or presence) *per se* of pets was associated with changes in any of the ADI-R social items. We selected two groups from the initial pool of 260 participants. The first group, G_alw,_ owned at least one pet at home since birth (n = 8; pets were dogs, cats and one rabbit). Among the G_alw_ individuals, three owned two pets. These G_alw_ individuals were matched with control individuals - who never owned a pet (G_0B_, n = 8) - for the same individual’s characteristics as in study 1 (all chi-square and Mann Whitney U-tests p>0.05; Table 1). Both the total score and the sub-scores of the ADI-R were not significantly different (all Mann-Whitney U-tests, p>0.05; Table 1). The G_alw_ and G_0B_ mean age was 11.1±1.9 years old at t_1_.

### Statistical Analyses

Changes between item scores at t_0_ and at t_1_ in each group (G_pet_, G_alw_, G_0A_ and G_0B_) were evaluated using Wilcoxon’s matched-pairs signed rank test. When a significant effect was observed, Mann-Whitney test was then applied to evaluate whether or not the change could be associated with the following variables:

• individual’s gender
• reasons for obtaining the pet(s)
• presence of different pets
• type of human-pet interactions (including privileged relationship)
• life setting (i.e. urban or rural)

Spearman’s rank order correlation assessed the correlation between the individual’s age or IQ score and his or her ADI-R item score. Since 36 tests were performed at both t_0_ and t_1_, in order to avoid false positive due to chance, Bonferroni correction for multiple comparison was applied systematically (p<0.0014).

## Results

### Study 1

**Figure 1:**
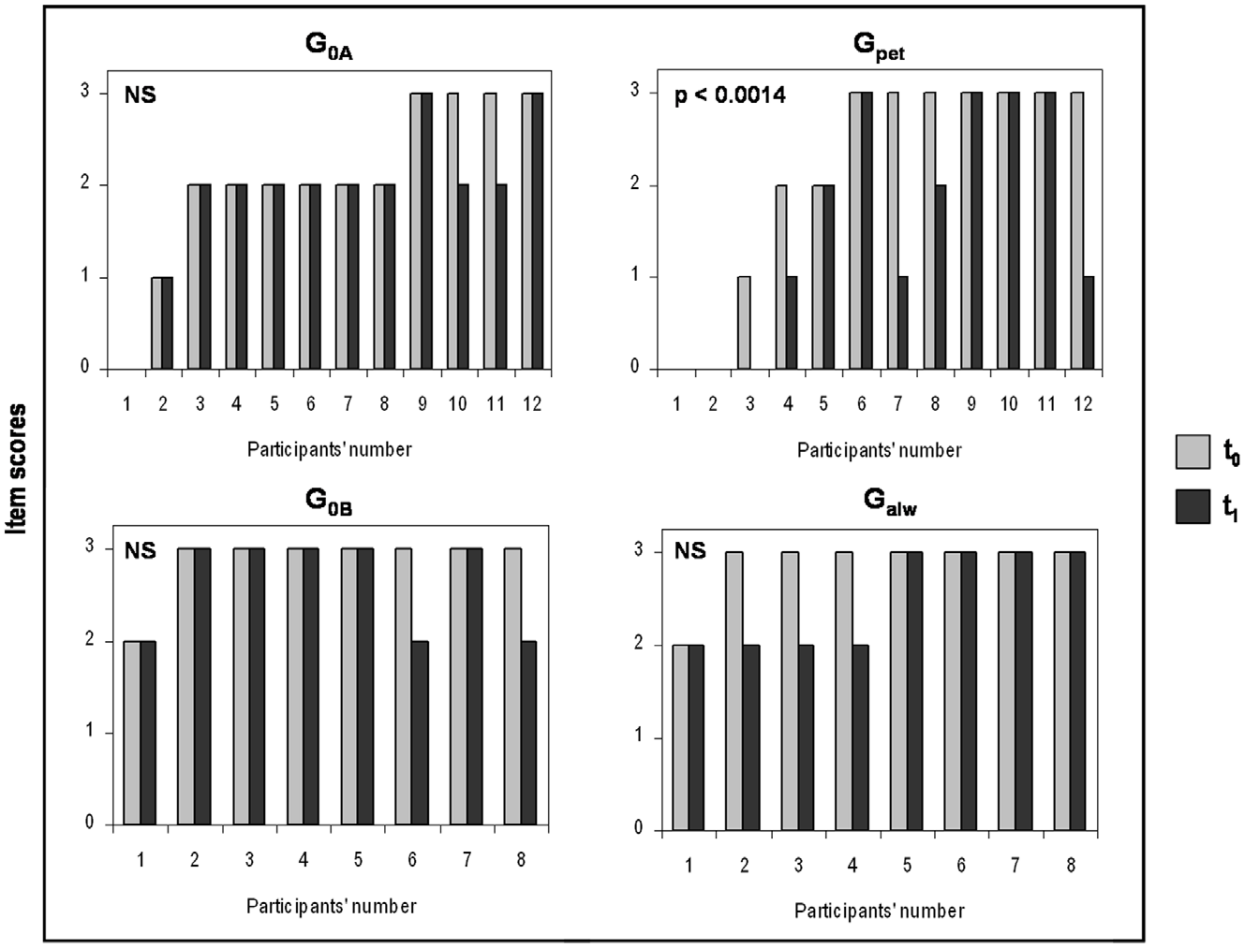
Item scores of “offering to share” at t_0_ (4-to-5-years old; in grey) and t_1_ (current period; mean age: 129.9±55.8 months old; in black) for G_0A_ (group with no pet in the family), G_pet_ (group with a pet arriving after the child’s 5^th^ birthday), G_0B_ (group with no pet in the family) and G_alw_ (group always with at least one pet at home since birth). Higher the score, more significant was the “offering to share” (e.g. sharing food or toys with parents or other children).

**Figure 2:**
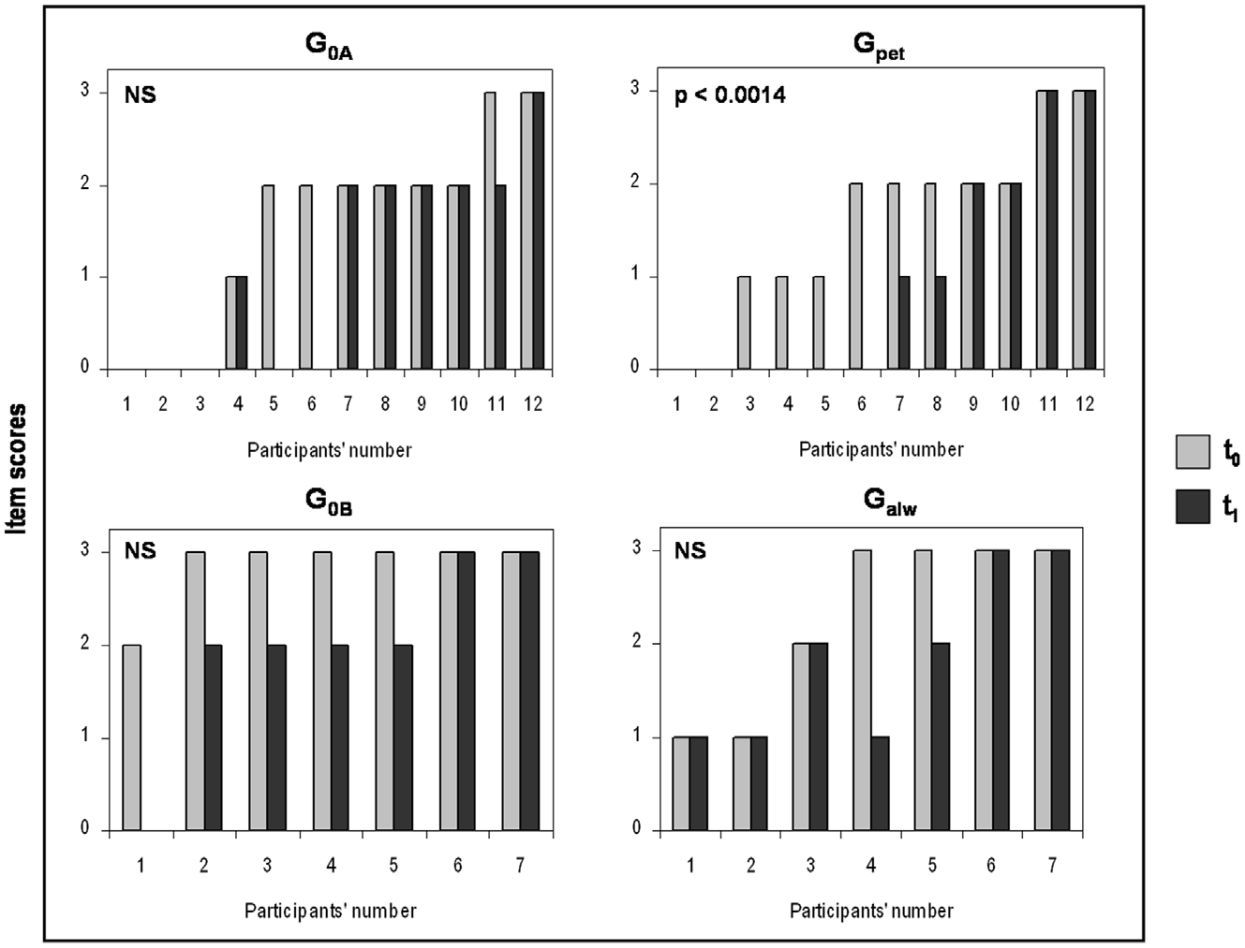
Item scores of “offering comfort” at t_0_ (4-to-5-years old; in grey) and t_1_ (current period, mean age: 129.9±55.8 months old; in black) for G_0A_ (group with no pet in the family), G_pet_ (group with a pet arriving after the child’s 5^th^ birthday), G_0B_ (group with no pet in the family) and G_alw_ (group always with at least one pet at home since birth). Higher the score, more significant was the impairment “offering comfort” (e.g. reassuring parents or peers who were sad or hurt). Comparisons were performed using Wilcoxon’s matched-pairs signed ranks tests (Significant threshold: p<0.0014).

Comparison of ADI-R assessment between t_0_ and t_1_ revealed significant changes in two of the 36 items in the G_pet_. Thus, G_pet_ had a lower deficit score for the items “offering to share”, e.g. sharing food or toys with parents or other children (Wilcoxon test: Z_Gpet_ = 21 p<0.0014; Fig. 1) and “offering comfort”, e.g. reassuring parents or peers who were sad or hurt (Wilcoxon test: Z_Gpet_ = 21 p<0.0014; Fig. 2). No changes were observed for the control individuals (Wilcoxon tests: Z_G0A_ = 3, Z_G0A_ = 6 p>0.05 in both cases; Fig. 1, Fig. 2). In G_pet_ and G_0A_, neither the total scores of ADI-R at t_0_ and t_1_ (Wilcoxon tests: Z_Gpet_ = 4 p = 0.011; Z_G0A_ = 15 p = 0.065) nor the sub-scores in the main domains (all Wilcoxon tests: p>0.0014) were statistically different at p<0.0014.

Score differences between t_0_ and t_1_ were neither correlated with individual’s age (all Spearman’s rank order correlation p>0.05) nor affected by gender, life setting, presence of different pets, and type of human-pet interaction (all Mann Whitney U-tests p>0.05). Interestingly, whether the parents had acquired the animal for their child or for the family revealed no significant difference in ADI-R scores (Mann Whitney U-test = 53.5 p>0.05), indicating that the results were not influenced by the parents expectations on the pet’s impact. In addition, communication and non-social aspects (*e.g.* scores for repetitive behavior and stereotyped patterns) were not affected by the pet’s arrival (all Wilcoxon tests p>0.05). No significant correlation (Spearman’s rank order correlation, p>0.05) between the items “offering comfort” or “offering to share” and IQ scores (verbal IQ, performance IQ and full IQ) was observed.

Parental questionnaire offered some information about the interaction type G_pet_ individual had with his or her pet (Table 2). Tactile interactions were the most reported (i.e. 75%; n = 9), followed by time spent with the pet (n = 8), play (n = 7) and visual interactions (n = 7). Care was the least reported item (n = 6). Thus, seven G_pet_ individuals were considered by their parents as having a privileged relationship with their pets. Among the five remaining individuals, three owned a cat and two owned a dog.

**Table 2:** Number of individuals with autism who display different types of relationships with their pet according to parents.

Presence of each item	G_pet_ (n = 12)	G_alw_ (n = 8)
Tactile interactions	9	2 [4]
Visual interactions	7	3 [5]
Play	7	0 [2]
Care	6	0 [0]
Time spent with pet	8	3 [3]
Privileged relationship	7	2 [2]

As three individuals of G_alw_ owned two pets, the first number showed the first pet’s answer and the second number in brackets showed the second pet’s answer.

### Study 2

No significant change was observed for individuals with autism who owned a pet since birth or for control individuals (G_alw_ and G_OB_; all Wilcoxon test p>0.05; Fig. 1, Fig. 2). In G_alw_ and G_OB_, neither the total scores of ADI-R at t_0_ and t_1_ (Wilcoxon tests: Z_Galw_ = 9 p = 0.447; Z_G0B_ = 11 p = 0.363) nor the sub-scores in the four domains (all Wilcoxon tests: p>0.05) were statistically different at p<0.0014.

Here again, an exploration of the parental questionnaire offered some information about the interaction type G_alw_ individual had with his or her pet. Few individuals were reported as interacting with their pets (Table 2). Care and play were not mentioned. Two individuals spent time with their pet, four had tactile interactions and five had visual interactions. Only three G_alw_ individuals were considered by their parents as having privileged relationships with their pets (i.e. three dogs). However, two of the three individuals who owned the same pet since birth, neither interacted nor bonded with it (i.e. all items were reported as absent).

## Discussion

Comparison of ADI-R assessment between G_pet_ and G_alw_ at two different time periods revealed significant changes in ADI-R scores only in the group experiencing the pet arrival in their homes. However, these changes were limited to two ADI-R items, “offering to share” and “offering comfort”. These findings suggest an improvement in prosocial behaviors of the individuals with autism. These prosocial behaviors are mainly impaired in individuals with autism [1], [52]. The absence of a significant correlation with IQ scores might imply that these changes were not related to the level of cognitive functioning. Interestingly, the individual-pet interactions (i.e. bonding) were more - qualitatively and quantitatively - reported in the case of pet arrival than pet presence since birth. To our knowledge, this is the first study showing an association between pet arrival and changes in prosocial behaviors. Our study follows the footsteps of the human-pet reports on the improvement of prosocial behaviors in individuals with typical development [53], [54].

### On the Significance of Changes

On the one hand, two main possible explanations could account for these findings.

First, parents may have acquired a pet because they believed that it would improve the prosocial behaviors of their children with autism. In this case, their responses to the ADI-R could be biased. The following findings strongly suggest that this was not the case:

1. Only 6 pets (of the 15 pets in Gpet) were acquired especially for the individuals with autism; the others were acquired for another family member. Changes in the prosocial behaviors were observed in both cases. Thus, these changes were not related to parental expectations.
2. This “pet study” (and its related questionnaire) began after the ADI-R completion. This suggests that the parents were not aware of the possible pet impact at t1.
3. Improvement was found only for two of the 36 items, further indicating the non-bias character of parent's responses.

The second explanation is that the arrival of a pet may have triggered a change in the individuals’ “perception of the social world”. Pets are supposed to enhance different skills in children with typical development such as self-esteem, socio-emotional development and empathy [32], [33], [54]. According to several authors, children with typical development seem to learn prosocial behaviors through their interactions with pets (e.g. sharing with and stroking the pet) [30], [55]. Could this also be the case for individuals with autism? Only observational studies can reveal how individuals with autism interact with their pet and whether somehow they develop skills to understand pet’s behaviors or needs [56].

On the other hand, it is not very surprising that other ADI-R items did not change, not even those related to the prosocial behaviors. Since verbal exchanges with pets are excluded, we would expect no changes in language skills whereas parents can indeed influence such skills [57]. Moreover, other studies confirm that animals neither influence motor skills nor reduce restricted behaviors in children with autism [16].

### Potential Mechanisms

Numerous theories have been proposed to explain the pet’s influence on human life (for a review, see [58]). Animals are animates, thus differ from inanimates in regard to many biological characteristics such as motion or sensory properties. Specifically, animates are beings that know, perceive, learn and think. These abilities make them appealing ([59] in [60]).

Friedman et al. [61] proposed the bio-psycho-social model that considers pets could reduce loneliness and thus could also be considered as “transitional objects” especially for the children [31], [62]. Pets may also be considered as “distracters”. Brickel [63] and more recently Odendaal [64] proposed to explain this phenomenon by the attention-shift theory. They stated that when a human is in a stressful situation, a pet seems to distract him/her from the anxiogenic stimulus (e.g. unknown situations in the case of people with autism). Animal’s presence triggers human’s attention-shift. Attention-shift offered by a pet under repeated exposure to a stressful situation, leads to a decrease in anxiety. Therefore, a family pet may also become a source and a center of attention that could be useful in individual’s learning.

On the one hand, the presence of a pet can have a direct influence. When a human and a pet are interacting, each partner uses signals emitted by the other to adjust their behavior: the behavior of one influences the response of the other (e.g. between a dog and a child [65]–[67]). A bond or a relationship emerges from these series of interactions where both partners have expectations on the next interaction on the basis of the previous ones [68]. Thus, as stated by Filiatre et al. [65] the pet’s behavior “could contribute to the acquisition by the child of a more structured and more socially efficient behavioral repertoire”. Moreover, the attitudes that children display towards pets have an impact on their prosocial and social behaviors [34], [69]. On the other hand, a pet can have an indirect influence on children through the family. Indeed numerous parents state that pets can be precious tools with which they educate their children [29], [70], [71]. For example, Beck et al [72] showed that an increased knowledge about wild birds after a ten-week educational home-based program for feeding was associated with parental involvement.

People with autism have been shown to be less sensitive to human voices [73] or faces [74] than to other environmental stimuli. To our knowledge, little is known about how they perceive animals' characteristics, but they are quite able to classify their animal preferences based on pictures [75]. Using a task based on sorting by preference, Celani [76] showed that children with autism chose pictures with an animal (e.g. dog, cat) rather than the ones with objects. At last, some authors explained that the affinity of people with autism for pets comes from animal’s multisensory characteristic. In addition, according to these authors, an animal’s behavior seems to be easier to decode and to predict than that of a human partner [14], [17].

### Pet Presence versus Arrival

One intriguing finding was that similar results were observed for the individuals who were in the presence of a pet from birth and those who never owned a pet. Changes were only observed in the group where the pet arrived after the age of 5. Different hypotheses are possible and are explored below.

When the pet was reported to be present since the individual’s birth, one would expect a cumulative effect of its presence. We cannot exclude this effect even if the ADI-R did not clearly explore it here (e.g. neither a too low nor a specific effect was explored by ADI-R items). However, we proposed an alternative explanation. Individuals with autism may usually avoid unfamiliar social partners and display diminished interest in novelty [1]. But under certain circumstances, children with autism prefer new stimuli rather than familiar ones [77]. The presence of a pet may be a mere “additional” element of the environment, therefore not attracting special attention. This is consistent with our parental questionnaire revealing that few of these individuals (G_alw_) developed a real bond with the pet in comparison to the other group (i.e. G_pet_, pet arrival). For example, only a quarter of G_alw_ individuals had a privileged relationship with their pet. The sole presence of the pet did not confer benefit for the individuals with autism. Such situation was previously reported in the children with typical development : the quality of relationship with their own pet appears to be a direct determinant of their socio-emotional development [33] and “pet bonding” is a stronger determinant of pet-associated benefits than the sole pet ownership [78]. If we take a look at the other side, the pet may also have formed a preferential bond with another member of the family and therefore been less demanding on the individual with autism.

The other non-exclusive possibility is that the arrival of a pet strengthens the cohesion of the family and increases the levels of interactions between their members. Pet’s arrival plays an even more important role in the lives of children who have inadequate or destructive family and social environments [79]. Most families acquiring a pet experienced an increase in quantity and quality of time spent together and felt happier after pet’s arrival [39]. This situation might be due to the collective attention on the new pet. This new pet arrival might induce an increased interest of the individuals towards the pet and/or their involvement in the family’s interactions. Cain [39] talked about the “triangling” process initiated by the pet (i.e. structuring and promoting interactions between two humans).

In our study, playing with the pet was reported by seven of the parents in G_pet_ whereas only two of the parents in G_alw_ noticed it. This behavior is a powerful means by which children master skills that are important for their development [80]. Playing with a pet is a complex behavior, sometimes involving object manipulation as a means for practice and mastery of action schemas (i.e. sensorimotor play) or child’s ability for mental representation. Thus, it provides a child with means of practicing and understanding the events of his or her social world (i.e. pretend play) [81]. These behaviors are not only observed in humans but also in human-pet interactions [55], [82]. Such interactions may have some positive outcome: playing with a dog during pet therapy had beneficial impact on hospitalized children [83]. This implies that playing with a pet may be beneficial to individuals with autism.

Interestingly, in our study, taking care of the pet was reported by half of the parents in G_pet_ whereas none of the parents in G_alw_ noticed it. Our finding infers the positive influence of pet arrival on parental support in the development of individuals with autism. Previous studies have shown that parents use pets to teach their children how to take care of pets by giving them age-appropriate tasks [29]. With parental support, the child involvement towards a pet may influence his/her socio-emotional development [84].

Finally individuals with autism may be sensitive to an overall change in their social sphere. Therefore the changes may be related merely to the overall family functioning rather than the sole pet arrival. This however would not explain why only two precise items were affected and not the others.

### Conclusion

This study reveals that in individuals with autism, pet arrival in the family setting may bring about changes in specific aspects of their socio-emotional development. It suggests the improvement of some prosocial behaviors in such individuals under certain circumstances. Thus, it offers a “window of opportunity” to future longitudinal developmental studies to further confirm these findings and explain their underlying mechanisms. Given the current state of knowledge, we suggest further research exploring our hypothesis on the association between the arrival of a new pet and the change in a family dynamic to evaluate the impact of another child’s arrival.

Our study has limitations that need to be noted. Both our study design and its lack of power (40 individuals from an initial cohort of 260 participants) didn’t allow us to clarify the exact role of pets in the families who already owned pets. Nevertheless these first results open interesting lines of research exploring the efficacy of animals employed in AAT settings. Further studies with larger sample sizes (e.g. including more control groups) are needed to clarify the exact role of pets in this context.
